# The Relationship between Balance, Muscles, and Anthropomorphic Features in Young Adults

**DOI:** 10.1155/2012/146063

**Published:** 2012-01-26

**Authors:** Ragıba Zagyapan, Cihan Iyem, Ayla Kurkcuoglu, Can Pelin, Mustafa Agah Tekindal

**Affiliations:** ^1^Department of Anatomy, Faculty of Medicine, Baskent University, Baglıca, 06530 Ankara, Turkey; ^2^Department of Biostatistics, Faculty of Medicine, Baskent University, Baglıca, 06530 Ankara, Turkey

## Abstract

Posture can be defined as the form of the body when sitting, walking, or standing. There would be no problem if muscles interact in harmony with musculoskeletal system or nervous system. Posture analysis is crucial for clinical assessments in physical medicine and rehabilitation. However, studies into this issue are limited. In this study, the relationship between static standing balance and anthropomorphic features in healthy subjects was investigated. The study was carried out with a total of 240 students at Baskent University (116 females, 124 males) aged between 18 and 25 years. Type of balance of the subjects was determined with lateral posture analysis. Additionally, muscle shortness tests, subcutaneous fat thickness, and waist and thigh circumference were measured. As the results of lateral posture analysis, 107 subjects (71 males, 36 females) were detected to have anterior balance, 89 (41 males, 48 females) posterior balance, and 44 (12 males, 32 girls) neutral balance. Values of waist circumference, thigh circumference, and waist/thigh ratio were compared with all three balance types. A statistically significant difference was detected between these values in the subjects who had anterior balance (*P* < 0.05). In conclusion, a significant relationship was detected between muscle shortness, waist and thigh circumferences, and postural balance type.

## 1. Introduction

Posture is one of the most important factors affecting physical and mental status of the individuals through their lives. Posture in humans is affected by different factors including familial factors, anatomical structural impairments, postural habits, and occupation [1]. According to the definition of Posture Committee of American Academy of Orthopedics in 1947, posture is the regular and balanced arrangement of skeletal components so as to preserve supportive structures of the body from injury and progressive deformation [1, 2]. Cailliet stated that “posture may be in question if static spinal configuration does not lead to fatigue, pain in a certain period and is with acceptable ranges aesthetically” [2]. Kapandji defined ideal posture as body's being in balance with minimal stress and loading and stated that spinal movement segment is a mechanical system composed of two adjacent vertebrae, intervertebral disks, ligaments, and facet joints. Anterior and posterior structures share the load on this segment as two columns. Anterior column is the main supportive structure. Anterior column plays a static role, and posterior column plays a dynamic role [3]. With a balanced posture, body and profund dorsal muscles may support the upper half of the body with the minimal muscle strength. When center of gravity slides forward due to impaired posture, dorsal muscles try to improve posture with more effort and provide a balanced position [4]. Ligaments and muscles should be in balance for a proper posture. Imbalance of impaired posture leads to fatigue, skeletal asymmetry, and pain with nociceptive stimuli. Muscles extremely strain in order to maintain abnormal posture. Spasm and pain emerge in time. Weight is distributed to all body parts, shock is absorbed, range of movement is preserved, and thereby movements needed for stability and mobility are controlled independently with a correct posture [5, 6]. Posture analysis is crucial for clinical assessments in physical medicine and rehabilitation. It is important to establish the relation between postural balance and anthropometric measurements and determine the postural deviation in developing treatment programs in clinic (postural scoliosis, increasing lumbar lordosis, straight back posture, and others) and evaluating the different deformities that may have occurred [7]. Comprehensive studies are limited incompatibly with the significance of the issue. Particularly, studies investigating the effectiveness of postural impairments and treatment modalities are severely limited.

In this study, it is aimed to investigate the relationship between standing static posture and anthropometric measurements, and thus we studied the relationship between standing static posture and anthropometric features.

## 2. Methods

In the study, type of balance in lateral posture analysis was investigated, and anthropometric measurements were conducted with 240 students (116 females, 124 males) of Baskent University aged between 18 and 25 years. This study was approved by Baskent University Institutional Review Board and Ethics Committee (Project no. KA 11/42) and supported by Baskent University Research Fund. Subjects who had any orthopedic problems or history of musculoskeletal system operations were excluded. A measure sensitive to 1 mm was used for anthropometric measurements, Holtain brand of skinfold caliper sensitive to 2 mm was used to measure fat, and a plumb-line was used for lateral static balance analysis [8].

### 2.1. Parameter Descriptions

#### 2.1.1. Posture Assessment (Lateral Analysis)

**Figure 1:**
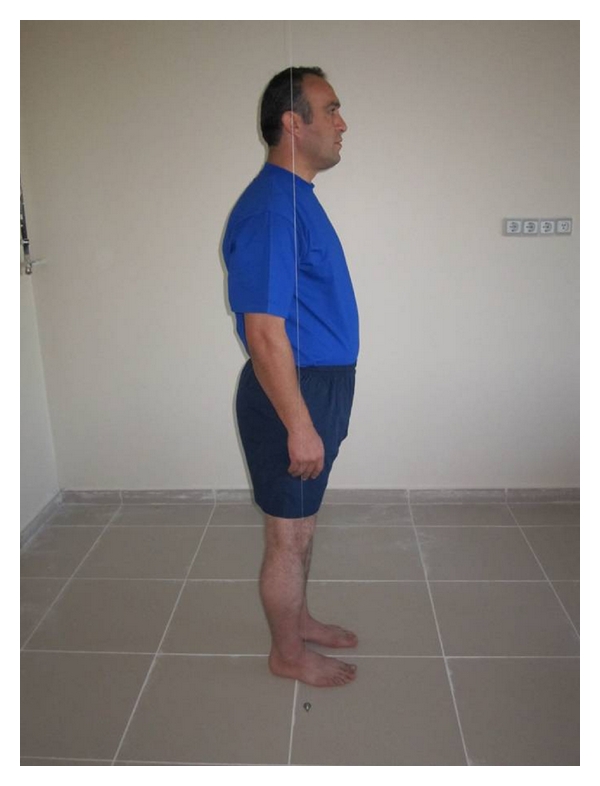
Postural assessment (lateral analysis) balance type.

Balance status was analysed with lateral analysis. A plumb-line hanged to the ceiling with a nylon string was used for this purpose. Anterior, neutral, or posterior balance status was determined according to the reference points through which the string passed from the ear lobe, shoulder joint, trochanter major, 1-2 cm anterior of knee joint, and just frontal section of lateral malleolus. As seen laterally from lateral, if reference points are in the anterior of the string, it was defined as anterior balance; if reference points are in the posterior of the string, it was defined as posterior balance; if the strings pass from reference points, it was defined as neutral balance (Figure 1).

#### 2.1.2. Measurement of Waist Circumference

Measurement was performed when the subject was standing and measurement area was determined using a 1 mm sensitive anthropometric measure by taking umbilicus as the reference point.

**Measurement of Thigh Circumference**

Measurement was done at the largest section of the thigh using a 1 mm sensitive anthropometric measure.

**Waist Circumference/Thigh Circumference Ratio**

Results of waist and thigh circumference measurements were proportioned. Whether there was a statistically significant relationship or not was analysed.

**Supraspinal Skinfold Thickness**

Thickness of skinfold between thumb and index finger at 5 cm superior and medial of spina iliaca anterior superior was measured with the Scinfold Caliper device.

#### 2.1.3. Muscle Shortness Tests

Length of muscles was tested according to anthropometric criteria. Names and test definitions of the muscles and muscle groups by which shortness test were performed according to these criteria were as follows.

**(a) Pectoral Muscles**

**Figure 2:**
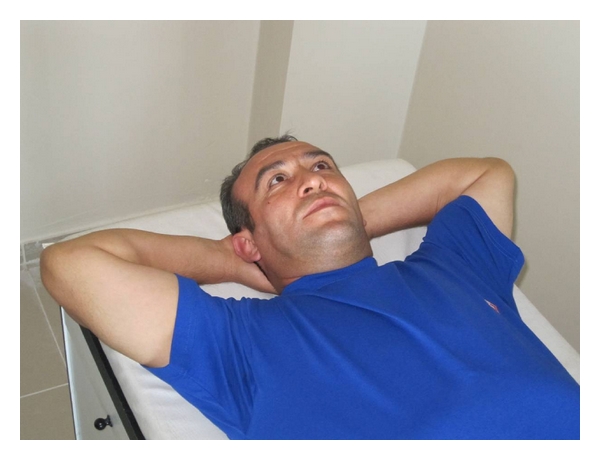
Pectoral muscles shortness test.

The subject was asked to put his/her hands at the back of the neck and arms were wanted to be loose when the subject was lying in supine position on the examination couch. If elbows are not to touch the couch, it is defined as muscle shortness (Figure 2).

**(b) Hamstring Muscles**

**Figure 3:**
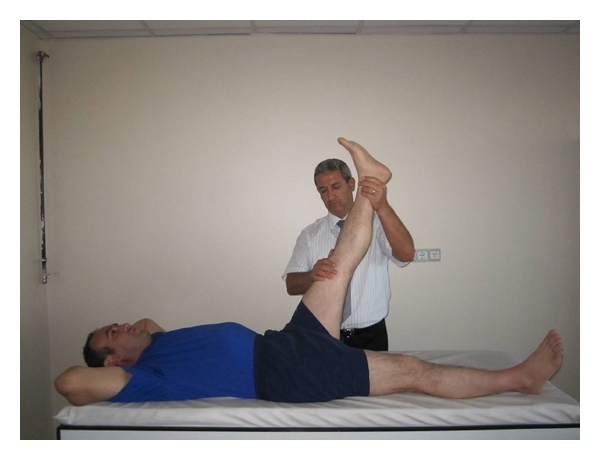
Hamstring muscles shortness test.

When the subject was in supine position on the examination couch, he/she upheld his/her lower extremities separately and knees were in extension. If the subject stated that he/she felt pain and strain in hamstring muscles, it would be defined as muscle shortness as well (Figure 3).

**(c) Gastrocnemius Muscles**

**Figure 4:**
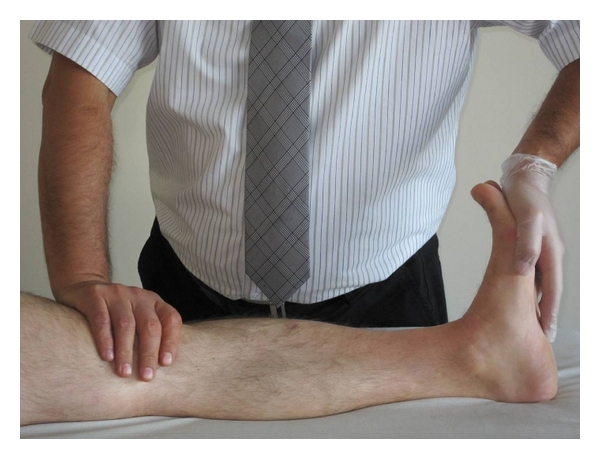
Gastrocnemius muscle shortness test.

When the subject was in supine position with knee extended on the examination couch, students were asked to bring his/her ankle joint to dorsiflexion. Inability to make dorsiflexion of the foot was defined as muscle shortness (Figure 4).

**(d) Lumbar Extensor Muscles**

**Figure 5:**
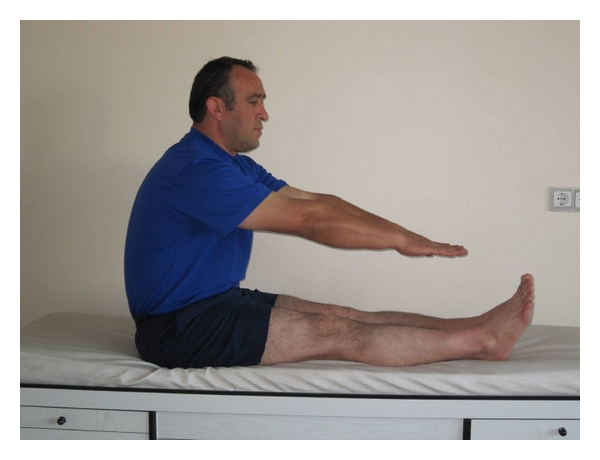
Lumbar extensor muscles shortness test.

When the subject was sitting with his/her legs outstretched, he/she was asked to touch tips of his/her fingers to toes. Inability to do this was defined as muscle shortness (Figure 5).

**(e) Hip Flexors**

**Figure 6:**
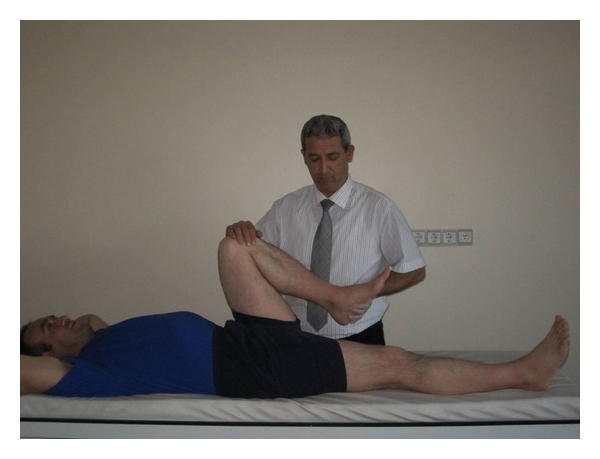
Hip flexor muscles shortness test.

When the subject was in supine position on the examination couch, if contralateral hip and knee come to some degree of flexion when lower extremities come to flexion from hip and knee joint, respectively, it was defined as shortness of the extremity (Figure 6).

## 3. Statistical Analysis

Age, height, weight, and body mass indexes were also evaluated in addition to parameters above. *t*-test was used for two groups, and one-way variance analysis was used for 3 or more groups. Chi-square analysis was used for determination of presence of a relationship and degree of relationship for continuous and discontinuous variables. Spearman correlation coefficients were estimated as *α* = 0.05 for all tests.

## 4. Results

Anterior balance was detected in 107 subjects (71 males, 36 females), posterior balance was detected in 89 subjects (41 males, 48 females), and neutral balance was detected in 44 subjects (12 males, 32 females) in lateral posture analyses. Three types of balance were compared in terms of mean values of waist circumference, thigh circumference, and waist circumference/thigh circumference ratio. A statistically significant difference was detected between them, mainly in anterior balance group (*P* < 0.05). Distribution between presence of shortness in hamstring group muscles and hip flexors and balance types was analysed. Shortness of these muscles showed statistically significant differences among all three balance types in the ones with anterior balance (*P* < 0.05). A statistically significant relationship could not be found between other parameters (lumbar extensors, supraspinal DKK, m. gastrocnemius, pectoral muscles) and balance types. Results of statistical analysis between body balance types and anthropomorphic features are shown in Table 1.

Results of correlation analysis between body balance types and anthropomorphic features are shown in Table 2.

## 5. Discussion

Anterior balance was more frequent among the subjects who had shortness in hamstring group muscles and hip flexors. Contrary to the expectations, a significant relationship could not be found between lumbar extensor shortness and posterior balance. Anterior balance was detected more frequent among the subjects with higher waist circumference, thigh circumference, and waist/thigh circumference. This was a result of forward change of gravity center. No study was encountered in the literature on the relationship between postural balance and anthropometric properties. In the study by Keionen et al. investigating the relationship between body movements in postural balance and anthropometric factors in 100 adults, they concluded that changes in body balance in standing position could not be explained with only anthropometric features; however anthropometric features should be emphasized in balance studies [9]. Study results indicate that height, weight, and emotional conditions could also be effective on balance. In a study of De Souza and Gil Coury conducted in Japan and Brazil, postural changes were investigated in 32 morbid obese patients and obesity was found to negatively affect anterior, posterior, and lateral balance and led to genu valgum deformity in 84.4% of the patients [10].

In our study, anterior balance was detected in 12 out of 14 (6 females, 8 males) subjects whose body mass index was 30 and above and hamstring and hip flexors were found to be shorter compared to normal subjects in terms of anthropometric values. This could be explained by the development of anterior balance resulting from the forward change of gravity center due to intense adipose tissue present around waist and belly in obese individuals. At the same time, shortness in hip flexor group muscles is a natural result in these individuals of anterior balance type. In a study of Gurfinkel et al. in USA, postural muscle tone in body axis was analysed in healthy individuals and they stated that postural changes caused alterations in length of axial and proximal muscles [11]. In our study, shortness was found in different muscle groups in the subjects who had different postural balances. Our results are consistent with the literature. Greve et al. investigated the relationship between dynamic balance and body mass index (BMI) in study conducted in Brazil and found a significant relationship between obesity and postural instability [12]. Postural instability was observed in obese individuals in the study. In their study, Csapo et al. compared 11 women who wear high-heeled shoes and 9 women who wear normal shoes and found shortness in gastrocnemius muscle and Achilles tendon compared to control group [13]. Study of Maribo et al. was carried out with 52 subjects with mechanic low back pain, and they suggested that mechanic low back pain had negative effects on postural control and caused pain in paravertebral muscles by altering center of gravity [14]. In their study, subjects were not asked about pain. However, these students daily spend ten hours at desk on average and high body part stays in flexion position. Therefore, habit of this standing position could explain the significantly high frequency of anterior balance in men. This situation is different in females, and higher frequency of posterior balance could be explained by the habit of wearing high-heeled shoes.

Leteneur et al. stated that forward and backward movements of the body at thoracolumbar region during walking affect the length of the muscles in hip group [15]. Similarly in our study, we stated that shortness of the muscles in hip flexor group was effective on anterior balance. Missaoui et al. stated that there was insufficiency in posture and balance among the patients with rheumatologic diseases and orthopedic problems. This condition was stated to be seen more frequently as the result of negative implementation of upper and lower extremities and vertebral column [16]. In a study by Horak, he reported three different approaches as functional, systemic, and posturographic for clinical evaluation of balance impairment. In the present study, measurements were also performed by function method. According to this method, the author concluded that there was impairment in biomechanical, motor, and sensory coordination in the ones with balance impairments [17].

The effects of postural muscle fatigue on the relationship between segmental posture and movement were investigated by Chabran et al., and they stated that a volunteer isometric contraction in upper extremity muscles did not cause fatigue in postural muscle groups [18]. In the current study, the authors can state that ideal balance posture was obtained with minimal contraction in postural muscle activation and there was a significant relationship between the changes in muscle length and balance type (anterior, posterior, and neutral).

Al-Khabbaz et al. reported that significant changes occured in body posture muscles and lower extremity muscles in the male university students who carried a backpack weighing more than 20% of their body weight and these changes had negative effects in providing control of postural balance [19]. In the study by Paillard was suggested that muscle fatigue-induced conduction impairment could emerge in motor and sensory components of postural control due to 25–30% loss of maximal volunteer contraction [20].

In light of literature findings and the results of this study the authors can conclude that there is a significant relationship between some anthropomorphic features (muscle shortness, waist and thigh circumference) and postural balance type. Finally, the authors consider that these results should be taken into account during the clinical assessments in the field of physical medicine and rehabilitation.

## 6. Conclusion

Postural analysis is important in physical medicine and rehabilitation fields for detecting and correcting postural deformities. In addition, postural evaluation is indicative in the treatment of short muscles determined in anthropometric measurements in healthy individuals and the resulting pain. For instance, hyperlordosis developing in lumbar region in people with posterior balance type could result in pain around waist. Similarly, flexor position of body in individuals of anterior balance type could cause pain in thoracal region based on kyphotic appearance. In postural evaluation, a weak relationship was reported between balance types and anthropometric properties.

 As a conclusion, there was a significant relationship between anthropometric properties (muscle shortness and waist/hip ratio) and postural balance type in the light of study results, and taking this information into account in the clinical evaluation of these individuals would be helpful for the treatment of painful standing defects.

## Figures and Tables

**Table 1:** Statistical analysis results between body balance types and anthropomorphic features.

Antrophomorphic features		Balance types			*P* < 0.05
		Anterior	Posterior	Neutral	
BMI (body mass index) (mean ± SD)		24.34 ± 4.26	23.29 ± 3.45	23.34 ± 3.71	0.127
Gender (N)	Male	71	41	12	**0.0001**
	Female	36	48	32	
Waist circumstance (mm)		839.87 ± 119.91	792.76 ± 108.06	797.70 ± 94.12	**0.008**
Hip circumstance (mm)		999.36 ± 85.85	969.78 ± 80.46	984.56 ± 68.65	**0.034**
Waist/hip circumstance (mm)		0.84 ± 0.07	0.82 ± 0.07	0.80 ± 0.06	**0.025**
Hip flexor muscles Shortness	Present	77	52	23	**0.031**
	Absent	30	37	21	
Lumbar extensor Muscles shortness	Present	80	63	26	0.158
	Absent	27	26	18	
Hamstring muscles Shortness	Present	62	38	16	**0.022**
	Absent	45	51	28	

**Table 2:** Relationship between balance types and anthropomorphic features.

Balance types Anterior = 1 Neutral = 2 Posterior = 3	Pectoral muscles shortness test		Hamstring muscles shortness test		Gastrocnemius muscles shortness test		Hip flexor muscles shortness test		Lumbar extensor muscles shortness test	Waist circumstance (mm)	Hip circumstance (mm)
	R	L	R	L	R	L	R	L			
*r* (Spearman Rank Correlation)	.159(*)	.151(*)	.145(*)	.145(*)	−.044	−.108	.132(*)	.133(*)	.047	−.192 (**)	−.127 (*)
*P*	.014	.019	.025	.025	.495	.095	.040	.039	.469	.003	.049
*N*	240	240	240	240	240	240	240	240	240	240	240

**Correlation is significant at the 0.01 level (2 tailed).

*Correlation is significant at the 0.05 level (2 tailed).
